# Cerium doping of 45S5 bioactive glass improves redox potential and cellular bioactivity

**DOI:** 10.1038/s41598-024-66417-y

**Published:** 2024-07-09

**Authors:** Jeong-Hyun Ryu, Tae-Yun Kang, Sung-Hwan Choi, Jae-Sung Kwon, Min-Ho Hong

**Affiliations:** 1 Department of Orthodontics, Institute of Craniofacial Deformity, Yonsei University College of Dentistry, Seoul, 03722 Republic of Korea; 2 Department and Research Institute for Dental Biomaterials and Bioengineering, Yonsei University College of Dentistry, Seoul, 03722 Republic of Korea; 3 BK21 FOUR Project, Yonsei University College of Dentistry, Seoul, 03722 Republic of Korea; 4 Department of Dental Biomaterials and Research Institute of Oral Science, College of Dentistry, Gangneung-Wonju National University, Gangneung, 25457 Republic of Korea

**Keywords:** Tissues, Biomedical engineering

## Abstract

45S5 Bioglass (BG) is composed of a glass network with silicate based on the component and can be doped with various therapeutic ions for the enhancement of hard tissue therapy. Nanoceria (CeO_2_) has been shown to indicate redox reaction and enhance the biological response. However, few studies focus on the proportion of CeO_2_-doped and its effect on the cellular bioactivity of CeO_2_-doped BG (CBG). In this study, we synthesized the CBG series with increasing amounts of doping CeO_2_ ranging (1 to 12) wt.%. The synthesized CBG series examined the characterization, mineralization capacity, and cellular activity against BG. Our results showed that the CBG series exhibited a glass structure and indicated the redox states between Ce^3+^ and Ce^4+^, thus they showed the antioxidant activity by characterization of Ce. The CBG series had a stable glass network structure similar to BG, which showed the preservation of bioactivity by exhibiting mineralization on the surface. In terms of biological response, although the CBG series showed the proliferative activity of pre-osteoblastic cells similar to BG, the CBG series augmented not only the alkaline phosphatase activity but also the osteogenic marker in the mRNA level. As stimulated the osteogenic activity, the CBG series improved the biomineralization. In conclusion, the CBG series might have a potential application for hard tissue therapeutic purposes.

## Introduction

In the dental and orthopedic field, synthetic bone substitutes (SBS) have been widely used over the past few decades as the regeneration of damaged hard tissue within the critical defects resulting from bone diseases such as fracture, trauma, or cancer^1^. The ideal SBS has crucial properties that include easy handling, biodegradability, biocompatibility, and osteogenesis^2^.

Bioactive glasses show as an attractive group, making them suitable for SBS due to the effective tissue response^3^. The discovery of the first BGs began with the development by L.L. Hench and colleague of 45S5 Bioglass (BG), which is composed of (45.0, 24.5, 24.5, and 6.0) wt.% SiO_2_, CaO, Na_2_O, and P_2_O_5_, respectively, which constituted the melt − quenching method^4^. The melt − quenching technique has been commonly used in the traditional melting approach that while offering easy processability for various sizes, is energy-intensive and time-consuming^5^. In contact with the body fluid at the surface of BG fabricated by the melt-quenching method, BG reacts at the surface forming the hydroxyapatite or hydroxyl carbonated apatite similar to the mineral phase of hard tissue, thus providing the strong bonding between materials and hard tissue^6^. Furthermore, it has been improved to liberate the ionic dissolution product through silicon (Si), calcium (Ca), and phosphorus (P) for supporting cellular activity^7^. For example, Westhauser et al. and Lopes et al. demonstrated the 45S5 BG enhanced the up-regulation of early osteogenic markers in vitro and increased the biomineralization by the ionic exchange in the physiological condition *in vivo*^8,9^.

BG is also favorable to the modification of its structure with helpful enhancement of metal oxide doping which allows the optimization of physicochemical properties by various clinical needs. Hence, the metal oxide-doped BG designed for hard tissue regeneration has been extensively researched. Numerous cations (e.g., Sr, Zn, Mg) have been developed in the 45S5 BG with enhancing osteogenic activity by proliferating the cells and expressing the osteogenic markers^10–12^.

Cerium (Ce) is a rare earth material that belongs to the lanthanide group. Ce is well-known to have attracted much attention in the biomaterials field through its interesting features owing to easily adjusting its electronic configuration to best fit its surrounding environment^13^. In particular, nanoceria has the anti-oxidant superoxide dismutase (SOD)-mimetic, catalase (CAT)-mimetic, and reactive oxygen species (ROS)-scavenging capability^14^. Specifically, nanoceria (CeO_2_) play a role in the protective cells against the damage resulting from the ROS which induces oxidative stress by generating the unstable radical oxygen owing to the changing oxidative state between Ce^3+^ and Ce^4+^ for redox reaction^15^. The reduction of excessive ROS from the damaged cells is necessary to preserve healthy biological functions. Considering the advantages of nanoceria, several studies have recently reported the nanoceria-doped BG^16–18^.

Although the advantages of doping CeO_2_ in the glass structure of BG are favorable for tissue regeneration, the acquiring maximal benefit is substantially dependent on the network structure from bioactive glass^19^. Glass network structure critically not only influences the solubility but also optimizes the releasing inducerons which favor cellular differentiation for new bone formation^20^. The optimized Ce content in BG can consequently affect the cellular response such as proliferation, differentiation, and mineralization as well as the performance of overall materials. Hence, it is significant to consider the effectivity of different Ce concentrations on the biological properties of BG.

**Figure 1:**
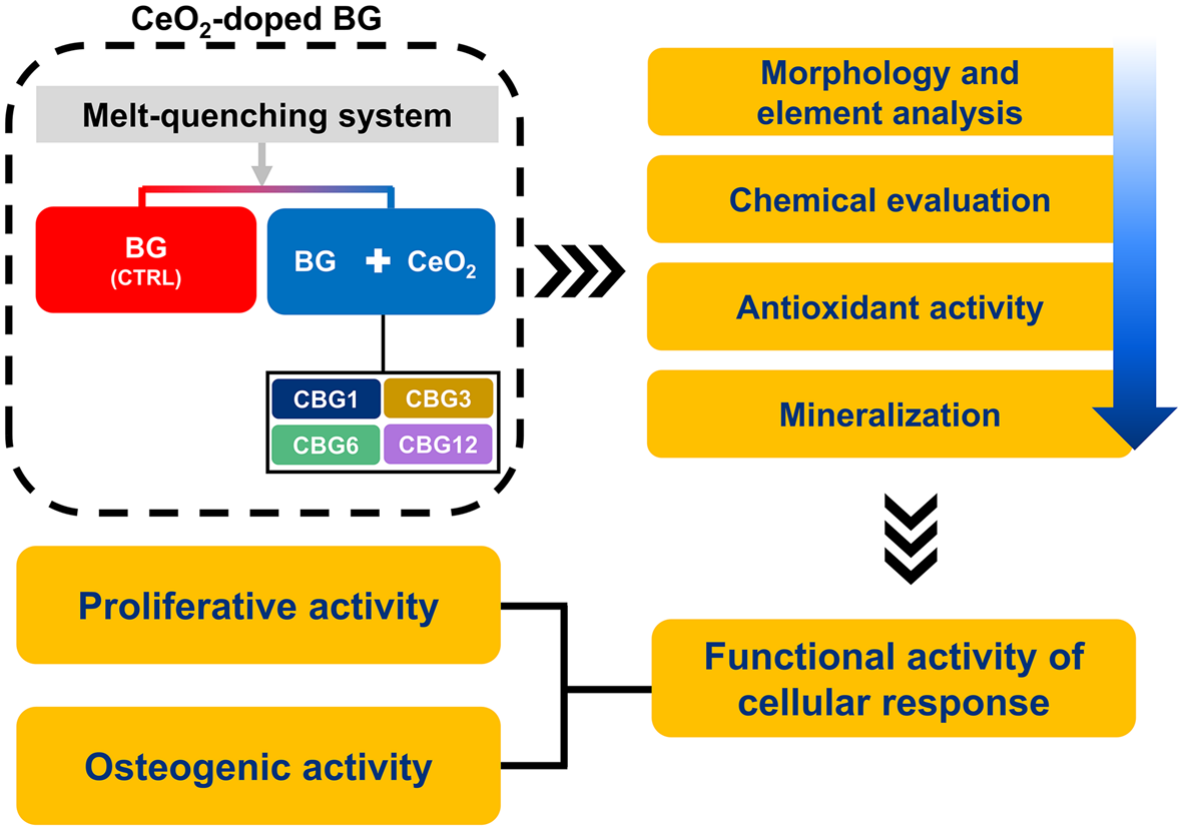
Flowchart showing the study design and experiment workflow.

This study aimed to synthesize CeO_2_-doped BG (CBG) with various concentrations of Ce and Na to determine the optimal structural composition that maximizes cellular activity. To attain this, we have synthesized CBG glasses with augmentation of Ce concentration in the glass structure to determine their effect on the bioactivity. We have also carried out the osteogenic response of the CBG glasses in vitro by evaluating their effects on cellular proliferation, differentiation, and mineralization. The results of our study can offer precious insight into the optimization of Ce concentration doped BG for use in dental and orthopedic applications. We exhibited the flow chart of this study as shown in Fig. 1.

## Results

### Physicochemical characterization of BG and CBG

#### Morphology and element composition of CBG as compared to BG

**Figure 2:**
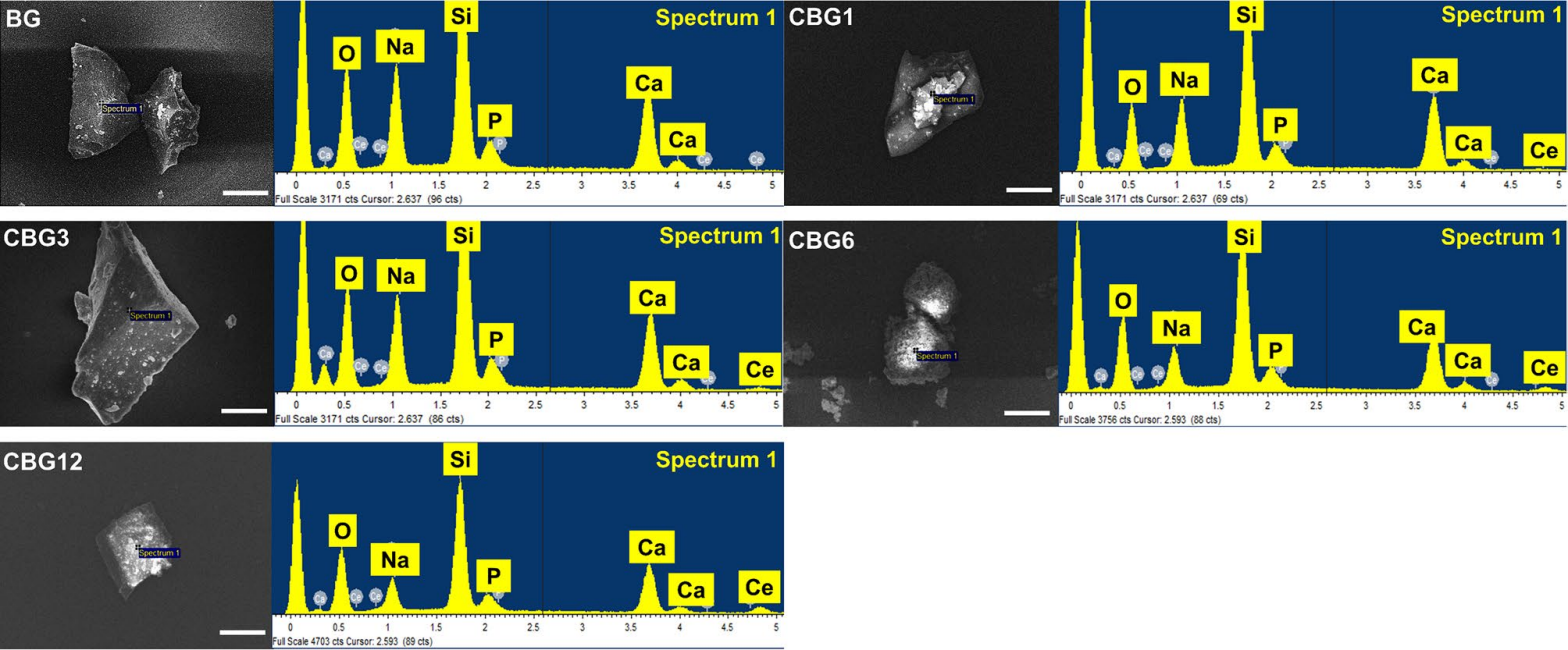
Morphology and element analysis of BG and CBG groups. Scale bar; 20 μm.

The surface morphology of glass particles was observed using SEM (Fig. 2). The SEM revealed glass particles with no significant difference in the morphology. The element analysis of the BG and CBG groups showed that there was no significant difference in the Si and Ca content. The relative composition of Na and Ce elements was consistent with the composition of their respective groups, with Ce levels increasing from BG to CBG12 groups (Supplementary Table 1).

### XPS of the oxidation states of Ce ions for CBG

**Figure 3:**
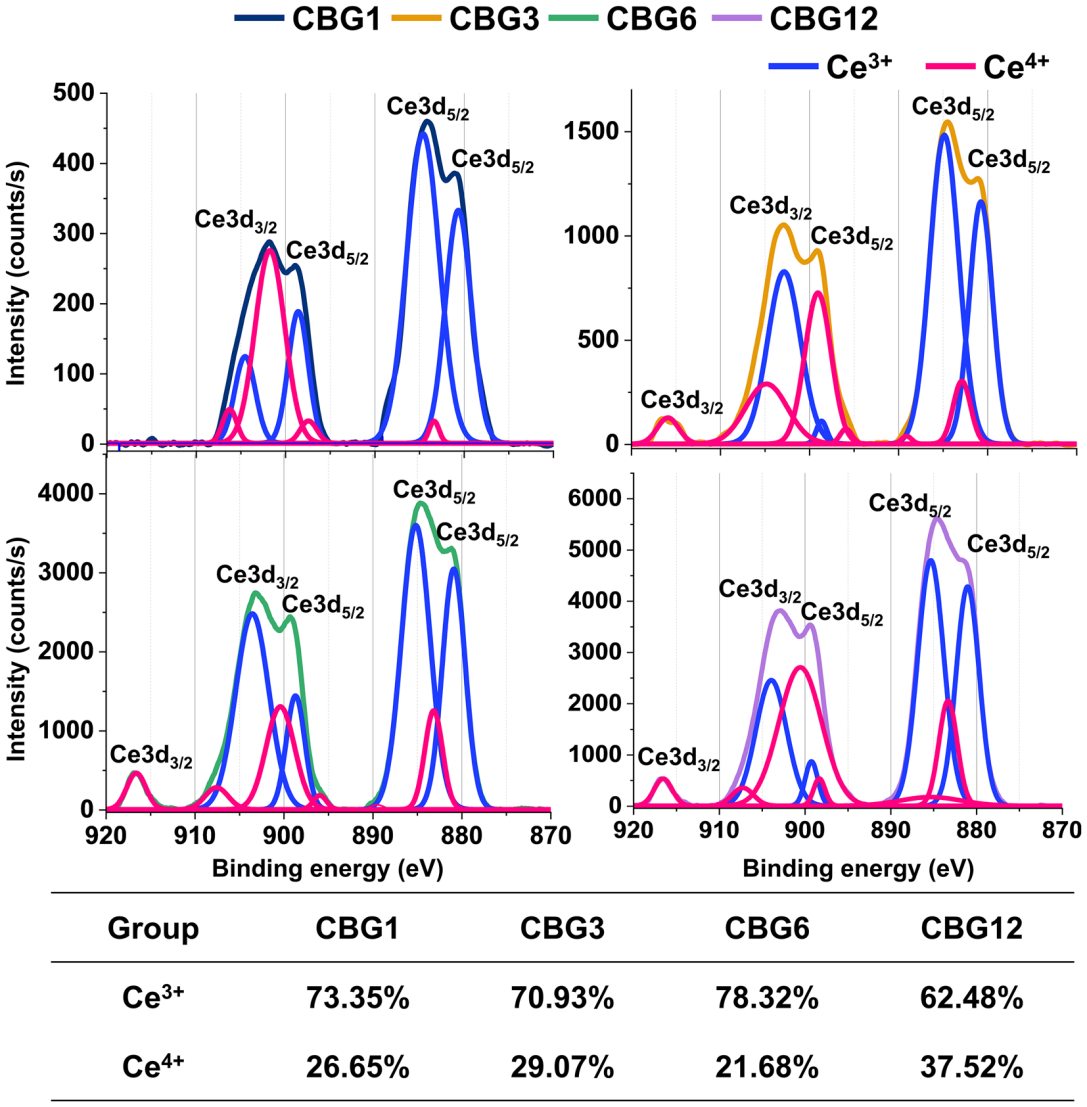
X-ray photoelectron spectroscopy survey spectra for CBG; Ce 3d deconvoluted photoelectron spectra from CBG1 to CBG12 groups.

To confirm the chemical environment and electron states, we observed using XPS spectrometric analysis. To investigate the redox state of Ce in the CBG group, we indicated the region of the XPS spectra characterization as a binding energy range of 880–917 eV (Fig. 3), where the inherent peak of Ce appears corresponding to emission from 3d levels. The Ce 3d core level involved two spin orbitals with 3d_5/2_ peak in the range of 882.6–898.5 eV and 3d_3/2_ peaks in the range of 901.2–919 eV. For analysis of Ce^3+^ and Ce^4+^ states by performing the deconvolution, both Ce^3+^ and Ce^4+^ oxidation states could be assigned to the spectra in agreement with the literature. The percentage of Ce^3+^ was increased as compared with that of Ce^4+^ until 6 wt.% CeO_2_ doped BG, while the percentage of Ce^4+^ state dramatically increased in 12 wt.% CeO_2_ doped BG as compared to the other groups.

### Structural phase of CBG as compared to BG

**Figure 4:**
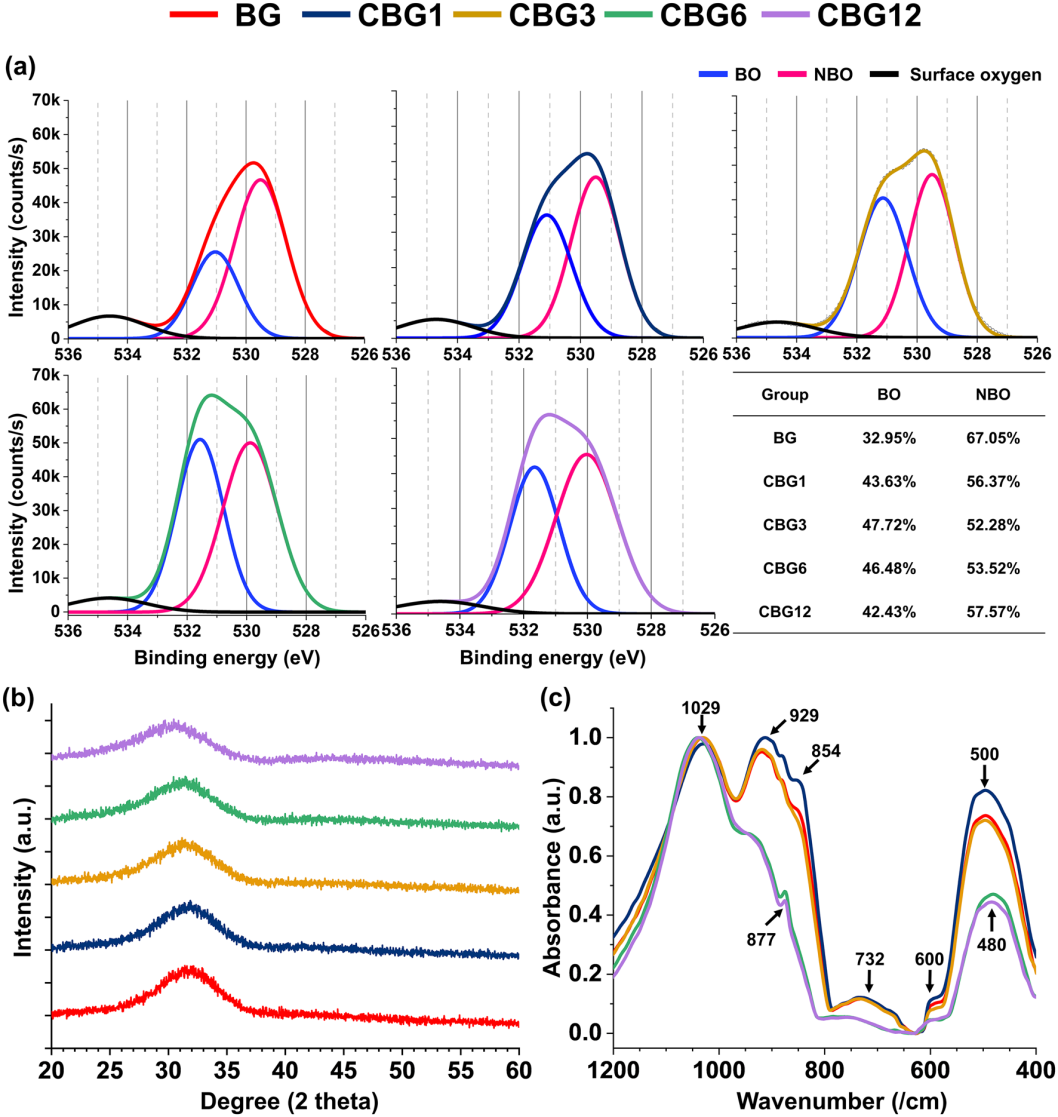
Physicochemical properties of CBG. (**a**) X-ray photoelectron spectroscopy for investigating the bridging oxygen and non-bridging oxygen, (**b**) X-ray diffraction pattern analysis for amorphous phase structure, and (**c**) FTIR analysis of the BG and CBG groups up to 12 wt.%.

Meanwhile, the glass network structure of BG and CBG groups was analyzed to the high-resolution O 1 s spectra for bridging oxygen (BO) and non-bridging oxygen (NBO) which indicated 532 eV and 530 eV, respectively (Fig. 4a). The range of BO was augmented by increasing the Ce levels in the CBG group as compared with the BG group, thus glass structure can be difficult to maintain the inherent of BG with increasing the Ce between 6 and 12 wt.%. As demonstrated by the glass structure phase, the XRD patterns of BG and CBG revealed a typical amorphous phase as a broad reflection from 20° to 40°, thus there were no obvious diffraction peaks in the XRD patterns (Fig. 4b). FTIR of 45SiO_2_-24.5CaO-(24.5-x)-6P_2_O_5_-xCeO_2_ series glass (with 0 < x ≤ 12) are shown in Fig. 4c. The FTIR spectra of the CBG1 and CBG3 groups showed similar vibration bands as compared with that of the BG group. The broad band at 500 cm^−1^ was indicated to be the combination of bending vibration of O–Si–O and O–P–O bands which are Q_si_^n^ and Q_P_^n^ units (where n represents BO, per SiO_4_ and PO_4_ tetrahedra, respectively). The broad band at 600 cm^−1^ was assigned to the P-O bending mode for amorphous phosphate. In terms of BO atoms in the silicate glass structure, the Si–O–Si symmetric stretching mode between tetrahedral structure indicated the broad band at 732 cm^−1^ and the asymmetric stretching vibration of Si–O–Si indicated the broad band at 929 and 1029 cm^−1^. On the other hand, the Si–O stretching of NBO atoms was attributed to the broad band at 854 cm^−1^.

Whereas, the FTIR spectra of the CBG6 and CBG12 group showed the different vibrations. The board band at 480 cm^−1^ was only assigned to Si–O bands and there was no observation of the board band at 600 and 732 cm^−1^ about the phosphate groups. Furthermore, the Si–O-Si symmetric stretching mode of board band at 1029 cm^−1^ was barely indicated and there was weakly indicated to the board band at 929 cm^−1^. Especially, CBG6 and CBG12 groups showed the band at 877 cm^−1^ that was attributed to Si–O–2NBO stretching mode.

### pH behavior and biodegradation of CBG as compared to BG

**Figure 5:**
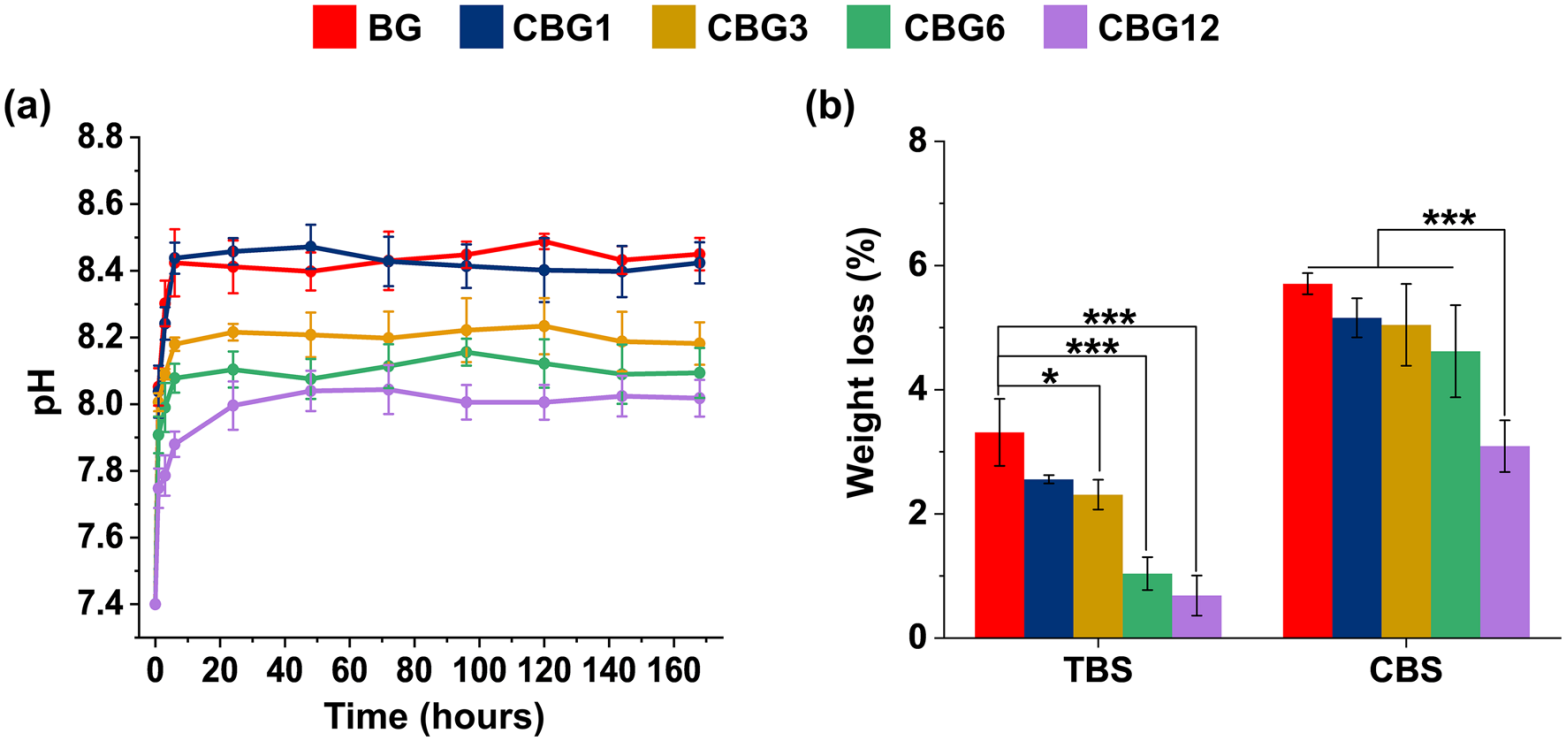
pH behavior and biodegradation rate of CBG groups against BG group. (**a**) observation of pH for 168 h, and (**b**) degradation rate in the neutral and acidic condition of CBG groups as compared with the BG group (*n* = 3; * *P* < 0.05; *** *P* < 0.001).

Considering the physiological environment where the materials will be exposed in clinical use, we observed the pH changes induced by CBG and BG groups as shown in Fig. 5a with Supplementary Tables 2 and 3. The CBG1 group exhibited a changed pattern of pH from approximately 7.4 to 8.4 similar to BG groups upon immersion in SBF for 6 h but there was not changed to the pattern of pH from 24 to 168 h. On the other hand, there was a reduction in the changed pattern of pH from CBG3 to CBG12 group as compared with BG groups for 6 h and maintained the pattern of pH from 24 to 168 h. Overall, the changed pattern of pH can be attributed to the augmentation of Ce levels and the reduction of Na levels as the different glass composition. As observing the degradation of the CBG group compared with the BG group for 5 days (Fig. 5b), the weight losses of cerium-containing glasses were reduced by increasing Ce content when compared to BG group and there was a significant difference as excepted by CBG1 group. On the other hand, in terms of acidic conditions, CBG12 groups significantly reduced the degradation as compared with the other groups, thus CBG12 groups had resistance to the degradation rate in both neutral and acidic conditions.

### Mineralization capacity of CBG as compared to BG

**Figure 6:**
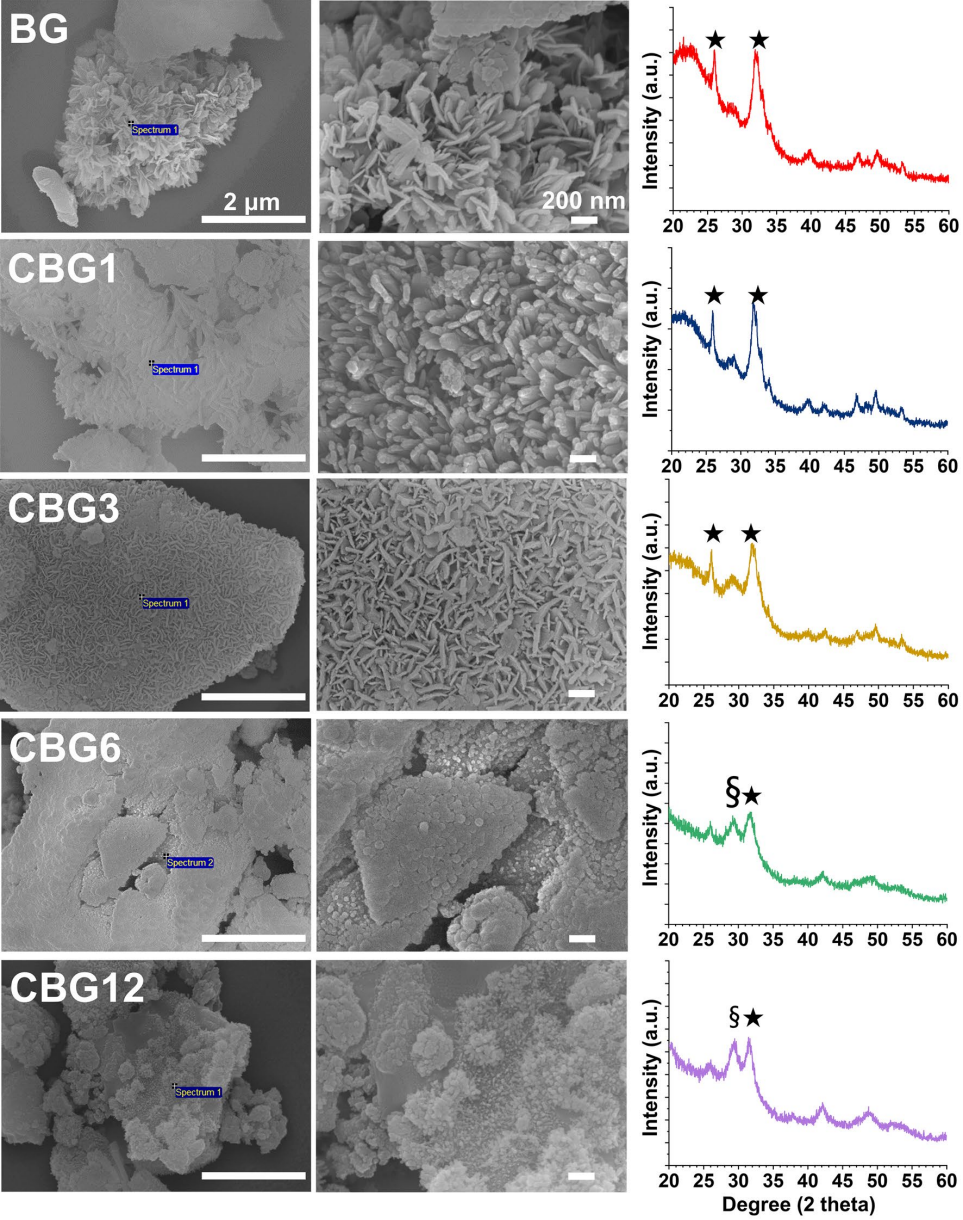
SEM images and XRD patterns of BG and CBG samples immersed in simulated body fluid for 14 days. BG and CBG groups showed the hydroxyapatite (HAp) peak according to JCPDS 09–0432. Notably, CBG1 was similar to BG with an indication of HAp. For CBG6 and CBG12 groups, there was a cerium phosphate peak by JCPDS 32-0199. ★, HAp peak; §, cerium phosphate peak.

CBG samples were immersed in SBF solution for 14 days. Surface morphology analysis and detection of components were then performed. Results are shown in Fig. 6 and Supplementary Table 4. The surface morphology of BG immersed in SBF for 14 days showed a hydroxyapatite (HAp)-like layer and agglomerated crystals. As compared with BG, the surfaces of CBG1 and CBG3 showed accumulation of the HAp-like crystal formation. In contrast, the surfaces of CBG6 and CBG12 were different in the accumulation of agglomerated crystals. Based on the results of SEM images, XRD analysis was performed due to confirmation of accumulated crystals in each group. BG, CBG1, and CBG3 groups showed approximately 26° and 32°, which were the first (211) and the second (002) signals of HAp by JCPDS 09-0432. On the contrary, patterns of CBG6 and CBG12 showed the HAp peak which indicated approximately 32°, while those of CBG6 and CBG12 exhibited the first (120) signal of cerium phosphate (CePO_4_) which indicated approximately 29° by JCPDS 32-0199.

### Antioxidant activity

#### SOD- and CAT-like activity of CBG as compared to BG

**Figure 7:**
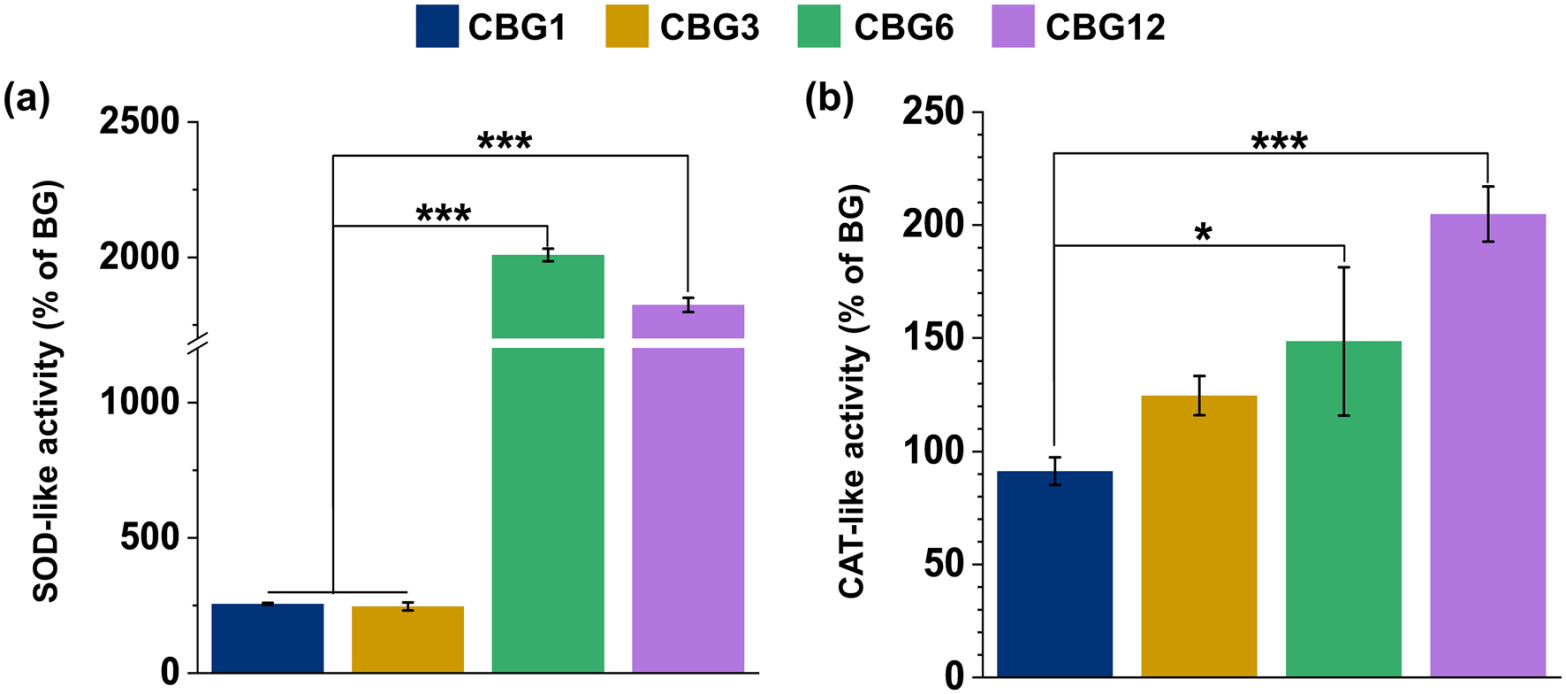
Antioxidant properties of CBG groups against BG. (**a**) SOD activity and (**b**) CAT activity of CBG groups as compared with BG (*n* = 3; *P < 0.05; ***P < 0.001).

As demonstrated by the antioxidant activity of CBG, we performed the SOD- and CAT-like activity for redox potential. In terms of SOD activity, CBG1 and CBG3 groups showed *ca.* (250 and 330) % augmentation, respectively, as compared with the BG group. On the other hand, CBG6 and CBG12 showed extreme augmentation of *ca.* (2,010, and 1,820) %, respectively, as compared to the BG group (Fig. 7a). Meanwhile, as compared with the BG group, CBG groups showed augmentation of *ca.* (91, 125, 148, and 204) % for CBG1, CBG3, CBG6, and CBG12 groups, respectively (Fig. 7b). We suggest the that CBG groups resulted in boosted antioxidant activity according to the augmentation of the Ce level incorporated in the BG.

### Biological activity

#### Mitochondrial metabolic activity and early osteogenic activity of CBG as compared to BG

**Figure 8:**
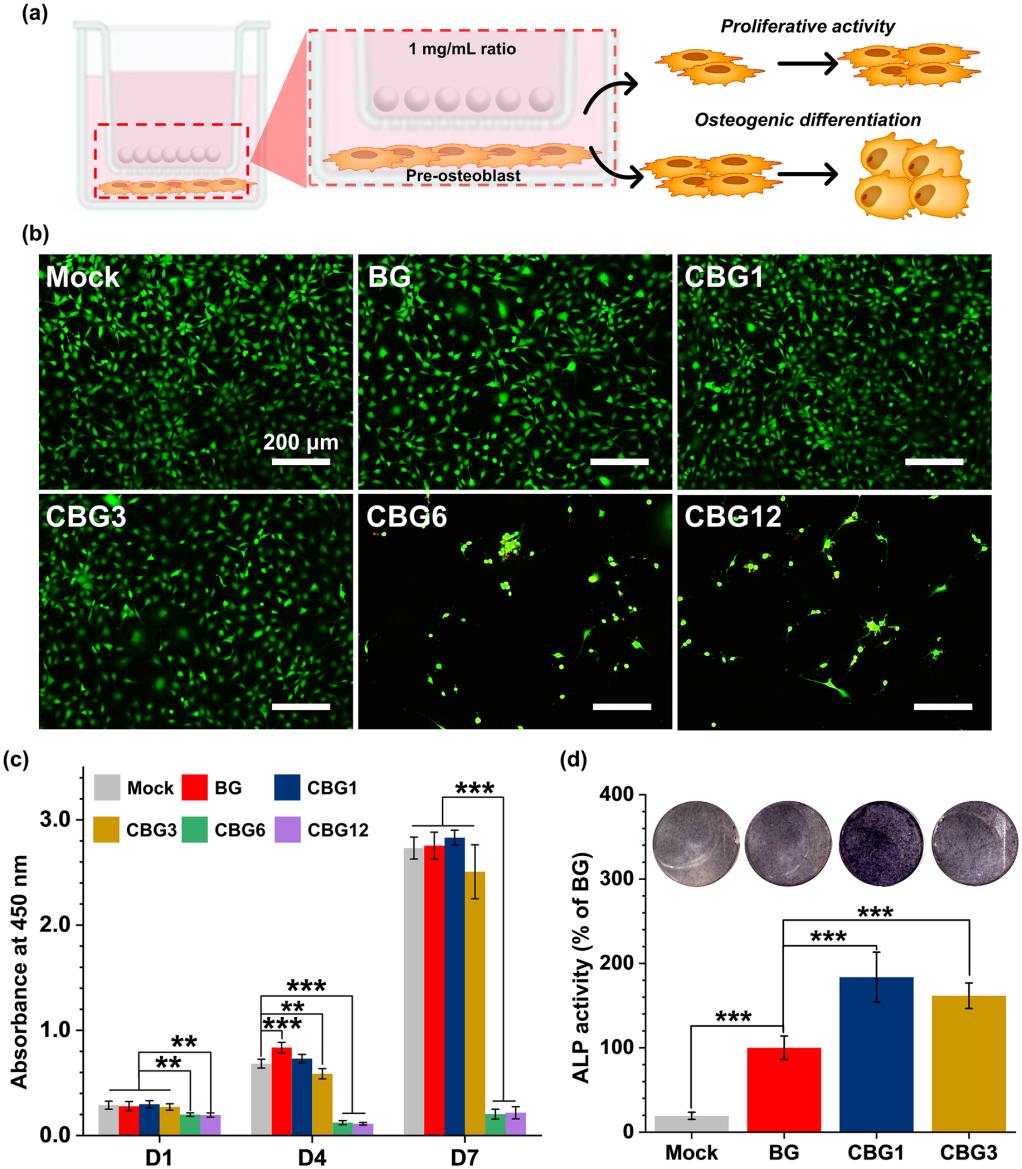
Mitochondrial metabolic activity and early osteogenic activity of CBG groups against BG group for 7 days. (**a**) Design of experimental conditions for measurement of cell proliferation and osteogenic differentiation for BG and CBG groups using by transwell. (**b**) Live/Dead assay for 24 h using by Calcein-AM (green) and ethidium homodimer-1 (red). (**c**) Mitochondrial metabolism activity of CBG groups against BG groups for 7 days. (**d**) Early osteogenic activity for qualitative and quantitative ALP of CBG groups against BG groups for 7 days (*n* = 3; **P < 0.01; ***P < 0.001).

Similar to the previous study^21^, we measured cell proliferation and osteogenic activity as shown in Fig. 8a. As a result, the cell attachment of MC3T3-E1 was observed in the CBG groups as compared with the BG group. Calcein-AM (green) selectively live cells and ethidium homodimer-1 (red) stained dead cells. Staining with live and dead cells was performed for 24 h, thus CBG1 and CBG3 groups showed a high density of live cells as compared with Mock and BG groups. On the contrary, the CBG6 and CBG12 groups showed a dramatically reduced amount of live cells as compared with the other groups (Fig. 8b). In terms of mitochondrial metabolic activity as proliferation, CBG1, and CBG3 groups reduced the proliferation of MC3T3-E1 as compared with BG groups, while CBG6 and CBG12 groups could not be proliferated for 4 days. For 7 days, BG, CBG1, and CBG3 groups had proliferative activity as excepted by CBG6 and CBG12 which caused the cytotoxicity (Fig. 8c).

For investigating the early osteogenic activity, qualitative and quantitative ALP measurements were observed for CBG groups against BG for 7 days. ALP staining of MC3T3-E1 in CBG1 groups became purple, darker than that in the other groups, with the darkest color being observed on the 7 days. The ALP activity was increased *ca.* 84% and 62% for CBG1 and CBG3 groups as compared with the BG group, respectively. The quantitative result of ALP activity was consistent with ALP staining (Fig. 8d). Furthermore, we carried out the observation of COL-I as an early osteogenic activity for 7 days (Supplementary Fig. 1). The intensity of COL-I expression in the MC3T3-E1 was augmented ca. 105% and 65% for CBG1 and CBG3 groups as compared with BG groups, respectively.

### Maturation of osteogenic activity and biomineralization of CBG as compared to BG

**Figure 9:**
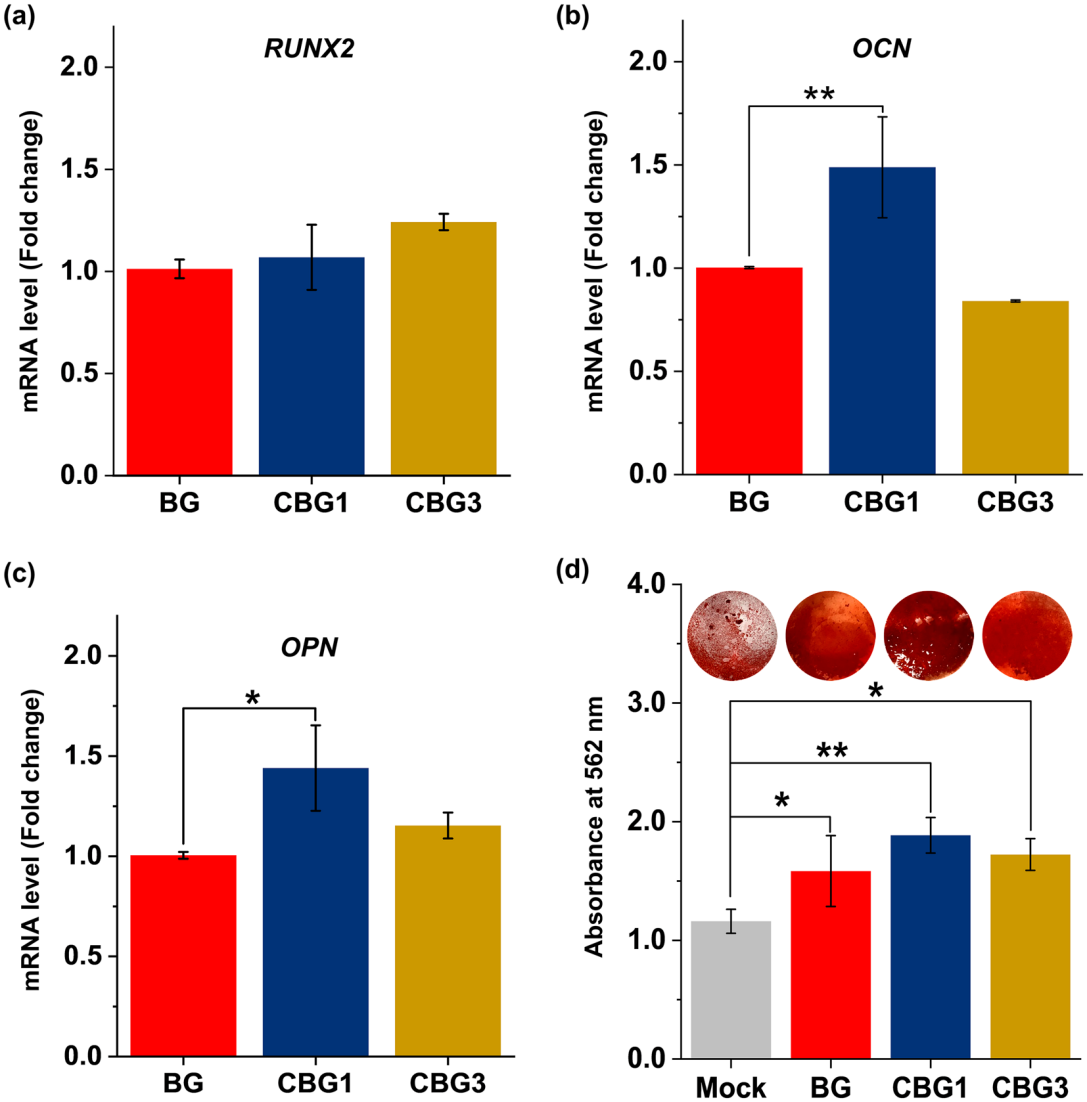
Osteogenic maturation and biomineralization of CBG against BG. (**a**) RUNX2, (**b**) OCN, and (**c**) OPN of qPCR analysis for 14 days and (**d**) biomineralization using by ARS for 21 days (*n* = 3; *P < 0.05, **P < 0.01).

To determine whether mature osteoblasts were obtained from pre-osteoblastic cells kept for 14 days in differentiating cell culture conditions, mRNA expression levels of RUNX2, OCN, and OPN as main osteogenic markers were investigated. On day 14, although RUNX2 expression levels were respectively augmentation by *ca.* 5% and 30% in CBG1 and CBG3 groups as compared with BG groups, there were no significant differences (Fig. 9a). Whereas, OCN and OPN associated with the mineralization phase showed the augmentation of *ca.* 75% and 60% in the CBG1 group as compared with the BG group, respectively (Fig. 9b,c).

Considering the enhancement of OCN and OPN mRNA levels as the main osteogenic markers, qualitative and quantitative biomineralization was shown in Fig. 9d. ARS staining of MC3T3-E1 in the CBG1 group became red, darker than that in the other groups, with the boldest red color being observed on the 21 days. The quantitative analysis of biomineralization revealed an increase of mineralization of *ca.* 36%, 62%, and 49% for BG, CBG1, and CBG3 groups as compared with the Mock group, respectively. Therefore, these indicated that the moderate optimal Ce from the CBG1 group was likely beneficial for enhancement of osteogenic activity.

## Discussion

BG has been widely used for therapeutic purposes as the construction of hard tissue such as bones and teeth. BG showed osteogenic differentiation as well as proliferative cellular activity by releasing the induceron (Si-Ca-P) from the glass network structure. For enhancing the osteogenic activity rather than BG, the various metallic or rare-earth atoms are incorporated in the BG by fabricating the melt-quenching method. Although the melt − quenching method as the common technique for the production of BG modulates the various metallic or rare-earth atoms, the solubility properties of the glass network structure need to be optimized. In the particular case of Ce, the fabrication of CeO_2_-doped BG (CBG) still faces the challenge that CeO_2_ possessing a high field strength has been aggregated for melting with other elements that incorporated the composition of BG. Considering the characterization of Ce when fabricating the CBG, Nicolini et al. and Malavasi et al. performed the physicochemical properties and cytotoxicity, but were limited to investigating the biological response for the optimal concentration of Ce^17,22^. Since there is no optimization of CBG for bioactivity and osteogenic activity, the optimal concentration of Ce is important to investigate the effects of biological responses such as proliferation and differentiation. Hence, we fabricated that the various series of CeO_2_ incorporated BG examine the physicochemical properties, antioxidant properties, and cellular activity for the optimal concentration of Ce.

Our findings revealed that the CBG group exhibited irregular particles, whereas no significant disparity was observed as compared to the BG group. However, elemental analysis of the CBG groups aligned with the respective group composition with increasing Ce levels. This observation is consistent with the prior literature. For example, Wang et al. synthesized CeO_2_-doped silicate bioactive glass using the melt-quenching method, yielding bulk glass particles exceeding 1 mm. They describe the augmentation of Ce levels in the silicate bioactive glass, while they did not investigate the morphology of glass particles^23^. Similarly, Placek et al. fabricated silicate–bioactive glass augmented with CeO_2_ via the melt-quenching method, resulting in microparticles smaller than 20 μm. They indicated that each glass particle was observed to have morphology consistent with augmenting the Ce atom level^24^.

Considering the oxidation state of Ce^3+^ and Ce^4+^ in the CBG groups, our XPS results showed that the percentage of Ce^3+^ was held rather constant in the range (70 to 78) %. Similarly, Nicolini et al. reported that BG incorporating CeO_2_ up to 5.3 mol.% via the melt-quenching method indicated the proportion of Ce^3+^ that remained relatively stable at approximately (73 to 76) %^25^. However, the CBG12 group showed augmentation of the Ce^4+^ oxidation state due to the higher temperature, which affected the melting isotherm and glass composition^26,27^. On the other hand, the XPS spectra of Ce3d indicated the oxidation state of Ce^3+^ and Ce^4+^. This is consistent with the previous studies. Atkinson et al. and Zheng et al. observed that the spectra of Ce3d could be assigned to both Ce^3+^ and Ce^4+^ oxidation states regardless of nanoceria content until 5 mol.% in the silicate bioactive glass^28,29^.

In terms of glass structure as XRD patterns, our XRD results showed the amorphous phase augmenting the CeO_2_ concentration at (1 to 12) wt.%. Similar to our XRD result, Leonelli et al. reported that BG in addition to CeO_2_ that was microparticle at (250 − 500) μm via the melt-quenching method indicated the amorphous phase, and did not negatively affect the glass structure, even at high concentration of CeO_2_^26^. However, we analyzed the surface of CBG groups for the presence of BO and NBO by XPS considering the glass network. Ce atom up to 3 wt.% concentration of CeO_2_ contributed to augmenting the BO in the glass network, while the concentration of CeO_2_ over 6 wt.% showed the reduction of BO in the glass network. Nicolini et al. reported that 5.3 mol.% CeO_2_-doped BG had a reduction of BO and augmentation of NBO due to the disruption of the glass former-based silicate and phosphate network in the glass network of BG^25^. Specifically, our FTIR results exhibited that the CeO_2_ ranging from 3 wt.% in the BG maintained the glass network former based on the BG, while a high concentration of CeO_2_ over 6 wt.% disrupted silicate and phosphate glass network due to the breaking Si–O–Si bonds and P − O bonds in terms of increasing the NBO. As expected, Nicolini et al. demonstrated that the Ce atom pushed Na atoms away from the phosphate group toward the silicate glass network, with the Ce atom playing a role in the network modifier with the competition of Na and Ca atoms, which can lead to depolymerization^25,30^.

In terms of interfacing the surface of the material for interaction with physiological fluid, SBF was used for the dissolution behavior examination, so that pH and weight loss were observed. Our dissolution results exhibited that pH and weight loss were significantly reduced by low solubility and chemical durability with augmentation of the Ce level. A study by Mostajeran et al. evaluating the weight loss when exposed to the phosphate-buffer saline at 37 °C demonstrated that the Ce-doped silicate-based bioactive glass indicated significantly low solubility, as compared with the Ce-free silicate-based bioactive glass, but they did not measure the pH^31^. Meanwhile, Kaur et al. suggested that the bioactive glass with Ce had greater chemical durability than the cerium-free bioactive glass, as demonstrated by the reduction of pH and low solubility, so that the Ce–O bond is covalent, and hydrolytic dissolution is difficult^32^.

On the other hand, our results indicated that CeO_2_ ranging from 3 wt.% doped into the BG accumulated the HAp formation on the glass, as compared with BG, while the concentration of CeO_2_ over 6 wt.% accumulated not only the HAp formation, but also the CePO_4_. In terms of the mechanism of bioactivity, hydrophilic NBO atoms and cations facilitate the bioactive process by opening the silicate network with favorable degradation through hydrolysis. This enhances the exchange of water molecules and SBF ions, which is consistent with the migration of Ca and phosphate ions on the glass surface, so that HAp was ultimately accumulated^33^. However, the higher content of Ce dopant can be caused by the release of Ce and phosphate ions from the samples in the SBF solution, so that Ce slows down the rate of HAp formation and accumulates the CePO_4_ on the glass surface, instead of HAp. In agreement with the literature, Leonelli et al. indicated that the augmentation of the CeO_2_ in the BG retarded the HAp formation due to the internal and external layers, indicating electrostatic interactions within the layer of silica gel and phosphate ion, thus the quick reaction on the glass surface accumulated CePO_4_^26^. A similar finding by Nicolini et al. evaluating the mineralization capacity when immersed in the SBF at 37 °C for 28 days demonstrated that the 5.3 mol.% Ce doped BG resulted in the augmentation of precipitation of CePO_4_, despite confirming the HAp formation^17^. Furthermore, Kaur et al. suggested that the augmented Ce atom had shown a reduction of negative charge on the glass surface that retarded the HAp formation, while accelerating the CePO_4_ formation^32^.

Meanwhile, the redox effect of Ce characterized by SOD- and CAT-like activity was evaluated. Regarding the cellular environment, the radical oxygen (^·^O_2_^-^) is a single molecule that generates the byproduct of normal cellular metabolism^34^. However, ^·^O_2_^-^ levels caused the production of cellular damages in response to noxious stimuli. Our SOD-like activity result showed that the high Ce concentration over 6 wt.% could scavenge ^·^O_2_^-^. As a result of scavenging ^·^O_2_^-^, H_2_O_2,_ and O_2_ are generated. The generated H_2_O_2_ is a reactive oxygen species and can be eliminated by catalase^17,34^. Our CAT-like activity results showed that the high concentration of Ce over 6 wt.% is suitable to remove the catalase.

In biological activity analysis, we investigated the proliferative activity of CBG groups for pre-osteoblastic cells. As a result of the cytotoxicity and proliferative activity, the concentration of Ce over 6 wt.% was deleterious to the damaged cells, and could not have proliferative activity. Placek et al. reported that the presence of 4 mol.% cerium-doped silicate-based bioactive glass led to the reduction of cell viability in osteoblastic cells, due to the dissolution products^24^. Additionally, Kutuldu et al. suggested that the amount of released Ce ions at such a high concentration in a dose-dependent manner induced cytotoxicity through mechanisms involving DNA damage and cell death, driven by augmentation of oxidative stress production^16^.

Considering the cytotoxicity of high concentration over 6 wt.%, we investigated the early osteogenic differentiation as ALP and COL-I. Both ALP and COL-I are important to promote the mature of extracellular matrix (ECM)^35,36^. As ALP and COL-I are expressed at the early stage of osteogenesis, they affect the maturation of ECM through the upregulation of OCN and OPN. OCN promotes calcium deposition by the regulation of calcium metabolism and OPN enhances the calcification in the ECM, thus OCN and OPN contributed to the maturation of osteoblast as a potential osteogenic marker for bone formation and remodeling process^37^. Interestingly, our results showed the concentration of Ce at 1 wt.% enhanced the osteogenic activity for upregulation of the osteoblast-specific gene (ALP, COL-I, OCN, and OPN) thus, biomineralization was promoted. The literature reported that the low concentration of Ce at 1 nM upregulated the osteogenic activity through the phosphorylation of Smad/1/5/8 protein in the bone morphogenetic protein signaling pathway and has the potential to enhance the osteogenic activity of the implant materials^38,39^.

In this study, we disclosed a favorable potential of CeO_2_ as a dopant in the BG to redox and enhance the osteogenic activity, as well as maintain the bioactivity of BG. For these advantages, cerium − containing BG could be applied to hard tissue regeneration as an enhancement of cellular activity and control to scavenge oxidative stress in tissue engineering and regenerative medicine. However, this study has some limitations. We should consider the ROS-induced cells, such as the treatment of hydrogen peroxide or lipopolysaccharide, to observe the redox states. Furthermore, we did not investigate the in vivo study as compared with BG for regenerative activity in a bone defect site. Considering these limitations, we should investigate the impact of Ce on redox states concerning ROS-induced pre-osteoblastic cells, and observe the regenerative activity of in vivo studies in future research.

## Conclusion

The present study demonstrates that doping Ce ranging from 3 wt.% in BG results in a composition with a stable glass network, good bioactivity in terms of HAp formation during the immersion in the SBF, redox potential, favorable cytocompatibility, and improved osteogenic activity. In contrast, the Ce concentration over 6 wt.% caused low bioactivity as CePO_4_ formation instead of HAp, and induced cytotoxicity, despite the high redox potential. Therefore, Ce doping can be controlled in the range of 3 wt.% in the BG, and might be applied to the bone grafting material for hard tissue regeneration. However, more studies are necessary to evaluate the osteogenic capability to treat hard tissue repair in vivo studies, such as the calvaria defect model.

## Methods

### Fabrication of cerium-substituted BG

Five compositions of BG were prepared by using reagent-grade chemicals in proportions appropriate for each case. In terms of the chemicals, SiO_2_ (Junsei Chemical Co., Tokyo, Japan), and CaCO_3_, Na_2_CO_3_, P_2_O_5_, and CeO_2_ (all Sigma–Aldrich, St. Louis, MO, United States) were used. The different compositions of BG were fabricated by melt-quenching methods according to the Table 1. Each batch was melted in a platinum crucible using an electric furnace (Lindberg, Asheville, NC, United States) at 1450 °C temperature for 1 h. The melted BG was then rapidly quenched in ice water to prevent crystallization and phase separation. The particles of BG were milled with a ball mill (Pulverisette 23, FRITSCH GmbH, Idar-Oberstein, Germany), and sieved through 400 mesh sieves (Gilson Co., Worthington, OH, United States).

**Table 1:** The composition of BG and CBG used in this study.

Code	SiO_2_	CaO	Na_2_O	P_2_O_5_	CeO_2_	Particle size (d_50_, μm)
BG	45.0	24.5	24.5	6.0	−	22.6
CBG1	45.0	24.5	23.5	6.0	1.0	27.8
CBG3	45.0	24.5	21.5	6.0	3.0	25.2
CBG6	45.0	24.5	18.5	6.0	6.0	21.4
CBG12	45.0	24.5	12.5	6.0	12.0	25.4

Unit: wt.%.

BG: 45S5 Bioglass®; CBG: Cerium-doped 45S5 bioactive glass, d_50_: glass particle median diameter size.

All experiments determined in this study were performed using glass particle of median diameter (d_50_) size from a laser diffraction particle size analyser (Mastersizer 2000, Malvern Ltd., Worcestershire, UK), except for the biodegradation analysis.

### Physicochemical characterization of CBG

The amorphous structure of the CBG powder was subjected to x-ray diffraction analysis (XRD, UltimaIV, Rigaku, Tokyo, Japan), using nickel filtered CuK_α_ radiation in the range of 2θ = 20 – 60° with a scanning speed of 1°/min and 0.02° step size. The functional group analysis of BG and CBG was performed by Fourier transform infrared spectroscopy (FTIR, Vertex-80 V/Hyperion 2000, Bruker, Breman, Germany), according to the previous literature^40^. The measurement of FTIR was carried out in the transmission mode within the range (400–1200) cm^−1^ and a resolution of 4 cm^−1^. The valance state of the elements in the BG and CBG powders was carried out using an X–ray photoelectron spectroscopy (XPS, K–alpha, Thermo Fisher Scientific, Waltham, MA, United States) analysis. A monochromatic Al–Kα (1486.6 eV) x–ray source was utilized. The percentage of Ce^3+^ and Ce^4+^ was calculated to peak areas from high resolution XPS scan of Ce3d core level. Furthermore, the percentage of bridging oxygen (BO) and non-bridging oxygen (NBO) was determined from peak areas under the O1s peak, respectively. To observe the morphology and detection of components, scanning electron microscopy and energy dispersive X-ray spectrometer (SEM–EDS, JSM-7800F, JEOL Ltd., Tokyo, Japan) was used to analyze the morphology and chemical compositions of BG and CBG. All of the samples were mounted onto the conductive stubs using carbon tape and sputter coated in platinum.

### Measurement of superoxide dismutase-like activity

The superoxide dismutase (SOD)-like activity was performed by using an EZ − SOD Assay kit (DoGenBio, Seoul, Republic of Korea) according to the manufacturer’s instructions. Briefly, the sample solution was immersed in deionized water to the ratio of 20 mg/ml at 37 °C for 1 h. To perform the SOD-like activity test, the samples and blanks were prepared as resumed in Table 2, and then incubated in the solution at 37 °C for 20 min. The SOD-like activity was determined by optical density at 450 nm. The inhibition rate (%) was calculated from the equation:

$$I.R(\%)=\frac{\left({OD}_{blank1}-{OD}_{blank2}\right)-({OD}_{sample}-{OD}_{blank2})}{\left({OD}_{blank1}-{OD}_{blank2}\right)}\times 100$$

where OD_blank1_, OD_blank2_, and OD_blank3_ are the absorbance at 450 nm of Blank1, Blank2, and Blank 3 sample and OD is the absorbance of sample.

**Table 2:** Preparation of the samples and blanks needed for the SOD mimetic activity test.

	Blank 1	Blank 2	Blank 3	Test sample
Sample	–	20 μL	–	20 μL
ddH_2_O	20 μL	–	20 μL	–
WST working solution	200 μL	200 μL	200 μL	200 μL
Dilution Buffer	–	20 μL	20 μL	–
Enzyme working solution	20 μL	–	–	20 μL
Total volume	240 μL	240 μL	240 μL	240 μL

### Measurement of catalase-like activity

Catalase (CAT)-like activity tests were performed using an Amplex-Red kit (Sigma–Aldrich, St. Louis, MO, United States) with a microplate reader. According to the manufacturer’s protocol, the dispersion of the samples was first incubated with 40 μM hydrogen peroxide, and then the mixture of Amplex–Red and horseradish peroxidase was added. The remaining H_2_O_2_ reacted with Amplex–Red to produce fluorescent resorufin, which was further measured at 560 nm. The CAT-like activity is defined as the percentage of H_2_O_2_ decomposed at the end of the assay.

### pH behavior

BG and CBG groups were immersed in the simulated body fluid (SBF; Biosesang, Seongnam-si, Republic of Korea). Briefly, 50 mg powders were immersed in 10 mL of SBF for 168 h at 37 °C in an orbital shaker (ISS-4075R, JEIO TECH Ltd., Seoul, Republic of Korea) with a shaking speed of 100 rpm. At each time point, the collected supernatant was measured by using a pH meter (Orion Star A211, ThermoFisher Scientific, Waltham, MA, United States).

### In vitro degradation

The in vitro degradation test was performed following by ISO 10993–14 considering the physiological condition. The particle of BG and CBG group was prepared from ranging of 315 to 400 μm. The bioactivity of samples was immersed in the Tris–HCl buffer solution (TBS) at pH (7.4 ± 0.1) by stimulating the body’s pH and citric acid buffer solution (CBS) at pH (3.0 ± 0.2) in an acidic environment at a ratio of (5.00 ± 0.05) g of glass to 100 ml of solution in an orbital shaker at 37 °C for 120 h with a shaking speed of 120 rpm. After 120 h, the samples were filtered from the solution by using a buchner funnel with filter paper (chm F1005, CHMLab group, Barcelona, Spain) and dried at 50 °C for 24 h to obtain a constant weight. The relative weight loss percentage (W_L_) of the samples after 120 h of immersion in solution was calculated from the equation:

$${\text{W}}_{{\text{L}}} \left( \% \right) \, = \, \left( {{\text{W}}_{0} - {\text{W}}_{{\text{t}}} } \right)/{\text{W}}_{0} \times { 1}00,$$

where W_0_ is the weight of the glass before immersion and W_t_ is the weight of the glass after immersion.

### In vitro bioactivity

To observe the apatite formation ability of CBG, the samples were immersed in SBF at a ratio of 1.5 mg/ml and incubated at 37 °C in an orbital shaker (120 rpm) for 14 days. After immersion of samples for 14 days, the samples were removed to the SBF and rinsed with deionized water and absolute ethanol. The rinsed samples were dried in the oven at 50 °C for 24 h. The dried samples were analyzed by SEM–EDS and XRD in the same condition as mentioned above.

### Cell culture

Cell culture was performed by using the mouse pre-osteoblastic cell line MC3T3-E1 (subclone 4, CRL-2593; ATCC, Manassas, VA, United States). The MC3T3 − E1 cells were cultured in alpha-modified minimum essential medium (α–MEM; Gibco, ThermoFisher Scientific, Waltham, MA, United States) supplemented with 10% Fetal bovine serum (FBS; Gibco, ThermoFisher Scientific, Waltham, MA, United States), and 1% penicillin/streptomycin (PS; Gibco, ThermoFisher Scientific, Waltham, MA, United States) (growth medium; GM) at 37 °C in a humidified incubator with 5% CO_2_. The osteogenic medium (OM) is based on the GM with 50 μg/mL L-ascorbic acid, 10 mM β–glycerol phosphate (Sigma–Aldrich), and 10 nM dexamethasone (all Sigma–Aldrich, St. Louis, MO, United States). The samples were placed in the cell culture insert (Millipore Corp., MA, United States) with a 1 mg/ml ratio, following the previous study^21^.

### Cytotoxicity assay

The MC3T3–E1 cells were seeded at 3 × 10^3^ cells in the 12-well plates and grown for 1 day. MC3T3–E1 cells were determined using the LIVE/DEAD Viability/Cytotoxicity kit (Thermo Fisher Scientific Inc) following the manufacturer’s instructions. Images were visualized using fluorescence microscopy (EVOS FL, Thermo Fisher Scientific Inc).

### Mitochondrial metabolic activity

Mitochondrial metabolic activity was performed by using MC3T3-E1 pre-osteoblastic cells with indirect contact methods. MC3T3-E1 cells were seeded at 3 × 10^3^ cells in 12-well plates and grown for 1, 4, and 7 days. Cell proliferation was determined by water-soluble tetrazolium salt assay (WST − 1, EZ − Cytox, DoGenBio, Seoul, Republic of Korea), following the manufacturer’s instructions. The absorbance of each well was measured at 450 nm using a microplate reader (BioTek, Winooski, VT, United States).

### Alkaline phosphatase staining and activity

To confirm the initial stage of differentiation for osteogenic activity, alkaline phosphatase (ALP) staining was performed. Briefly, cells were seeded at a density of 1 × 10^5^ cells in 24-well plates and replaced the OM. After culturing for 7 days, the cells were fixed by 4% paraformaldehyde (Biosesang, Seongnam-si, Republic of Korea) for 5 min. The fixed cells were rinsed to distilled water twice and treated to the SIGMA FAST BCIP/NBT ALP substrate (Sigma–Aldrich, St. Louis, MO, United States) for 1 h.

For measurement of ALP activity, cells were seeded at a density of 1 × 10^5^ cells in 6-well plates and cultured for 7 days. The cell lysates were obtained for ALP activity using a SensoLyte pNPP alkaline phosphatase assay kit (AnaSpec, An Jose, CA, United States) according to the manufacturer’s instructions. The total protein content was quantified by Pierce BCA protein assay kit (ThermoFisher Scientific, Waltham, MA, United States).

### Immunocytochemistry

To observe the early stage of differentiation for osteogenic activity, type I collagen (COL-I) was detected by the immunofluorescence staining. Briefly, cells were seeded on cover glass at a density of 3 × 10^3^ cells/well in 12‐well plates and replaced the OM. After culturing for 7 days, cells were fixed with 4% paraformaldehyde phosphate buffer solution (Wako Pure Chemical Corporation, Osaka, Japan). The fixed cells were rinsed with PBS, permeabilized with 0.1% Triton X-100 (Sigma–Aldrich, St. Louis, MO, United States), and blocked using 1% bovine serum albumin for 30 min at 25 °C. After blocking, the cells were incubated for overnight at 4 °C with COL-I primary antibody (1:500, Santa Cruz Biotechnology, Santa Cruz, CA, United States) in the dark. After incubation, the cells were rinsed with PBS and incubated in a secondary antibody (Alexa Fluor 488 conjugated Goat and anti-mouse IgG H&L, ThermoFisher Scientific, Waltham, MA, United States). After 1 h, all samples were rinsed with SantaPBS and finally mounted on a microscopy glass using ProLong Gold Antifade Mountant with DAPI (4’,6-diamidino-2-phenylindole; ThermoFisher Scientific, Waltham, MA, United States). The sample images were observed using the confocal fluorescence microscope (LSM 900, Carl Zeiss, Thornwood, NY, United States). The recorded images were analyzed, and the fluorescence intensity was quantified (geometric mean) using the software Zen (version 3.4, Blue edition, Carl Zeiss, Jena, Germany) in accordance with the previous study^41^.

### Quantitative polymerase chain reaction

MC3T3-E1 cells were seeded at the density of 1  × 10^5^ cells/well in the 6-well plates and cultured for 14 days. Total RNA was isolated by using trizol (Qiagen, Hilden, Germany) according to the manufacturer’s instructions. cDNA was synthesized from 1 ug total RNA by using PrimeScript RT reagent kit (Takara Biotechnology Co. Ltd., Tokyo, Japan). qPCR was performed with SYBR Green Reagent (Takara Biotechnology Co. Ltd., Tokyo, Japan) on the Quantstudio 3.0 Real-Time PCR system (Applied Biosystems, Waltham, MA, United States). The quantitative polymerase chain reaction (qPCR) amplification conditions were: 95 °C for 30 s, followed by 40 cycles of 95 °C for 5 s and 60 °C for 30 s. The following primer sequences were used in Table 3. The glyceraldehyde 3-phosphate dehydrogenase (GAPDH) was normalized in the relative gene expression level and calculated by using the 2^−ΔΔCT^ method.

**Table 3:** Primer sequences used for gene expression analysis.

Molecules	Primer sequence (5’-3’)	Product size (bp)	Accession No
RUNX2	Forward: GGGAACCAAGAAGGCACAGA Reverse: ACTTGGTGCAGAGTTCAGGG	152	NM_001271627.1
OPN	Forward: GAGGAAACCAGCCAAGGACTAA Reverse: TCTGGGTGCAGGCTGTAAA	140	NM_009263.3
OCN	Forward: TTGGCCCAdGACCTAGCAGA Reverse: CTGGGCTTGGCATCTGTGA	100	NM_007541.3
GAPDH	Forward: CCCACTCTTCCACCTTCGATG Reverse: CGAGTTGGGATAGGGCCTCT	201	NM_001289726.1

*RUNX2,* Runt-related transcription factor-2; *OPN*, Osteopontin; *OCN,* Osteocalcin; *GAPDH,* Glyceraldehyde3-phosphate dehydrogenase.

### Biomineralization assay

For the biomineralization, MC3T3-E1 cells were seeded at the density of 1 × 10^5^ cells/well in the 24-well plates and cultured for 21 days. The cells were fixed by 70% EtOH for 1 h at 4 °C. The fixed cells were rinsed with distilled water and stained in 40 mM Alizarin Red S (ARS) staining solution (pH 4.2, adjusted with 28% NH_4_OH (Sigma–Aldrich, St. Louis, MO, United States) for 15 min. After observation of each sample, ARS was diluted in 10 mM sodium phosphate (pH 7.0) containing 10% (w/v) cetylpyridinium chloride (Sigma–Aldrich, St. Louis, MO, United States) to quantify mineralized nodules. The optical density was measured at 562 nm by microplate readers (BioTek, Winooski, VT, United States).

### Statistical analysis

All data was presented in the form of the mean ± standard deviation in triplicate. Statistical analysis was performed by using a one-way analysis of variance followed by Tukey’s post hoc analysis and paired t-test analysis (SPSS26, Chicago, IL, United States). *P* value of 0.05 was statistically considered significant.

### Supplementary Information

Supplementary Information.

## Data Availability

Sequence data that support the findings of this study have been deposited in the National Center for Biotechnology Information with the primary accession number NM_001271627.1, NM_009263.3, NM_007541.3, and NM_001289726.1.
