# Supplementary material for: Cerium doping of 45S5 bioactive glass improves redox potential and cellular bioactivity
Source: Sci Rep. 2024 Jul 9;14:15837. doi: 10.1038/s41598-024-66417-y (PMC11233629; doi:10.1038/s41598-024-66417-y)
Supplement: Supplementary file 1 — Supplementary Information. [file 41598_2024_66417_MOESM1_ESM.docx]

**Cerium doping of 45S5 bioactive glass improves redox potential and cellular bioactivity**

**Jeong-Hyun Ryu^a,#^, Tae-Yun Kang^b,#^, Sung-Hwan Choi^a^, Jae-Sung Kwon^b,c,^*, Min-Ho Hong^d,^***

^a^ Department of Orthodontics, Institute of Craniofacial Deformity, Yonsei University College of Dentistry, Seoul 03722, Republic of Korea

^b^ Department and Research Institute for Dental Biomaterials and Bioengineering, Yonsei University College of Dentistry, Seoul 03722, Republic of Korea

^c^ BK21 FOUR Project, Yonsei University College of Dentistry, Seoul 03722, Republic of Korea

^d^ Department of Dental Biomaterials and Research Institute of Oral Science, College of Dentistry, Gangneung-Wonju National University, Gangneung, 25457, Republic of Korea

^#^These authors contributed equally to this work.

* Corresponding Authors to Jae-Sung Kwon (jkwon@yuhs.ac) and Min-Ho Hong (mhong@gwnu.ac.kr).


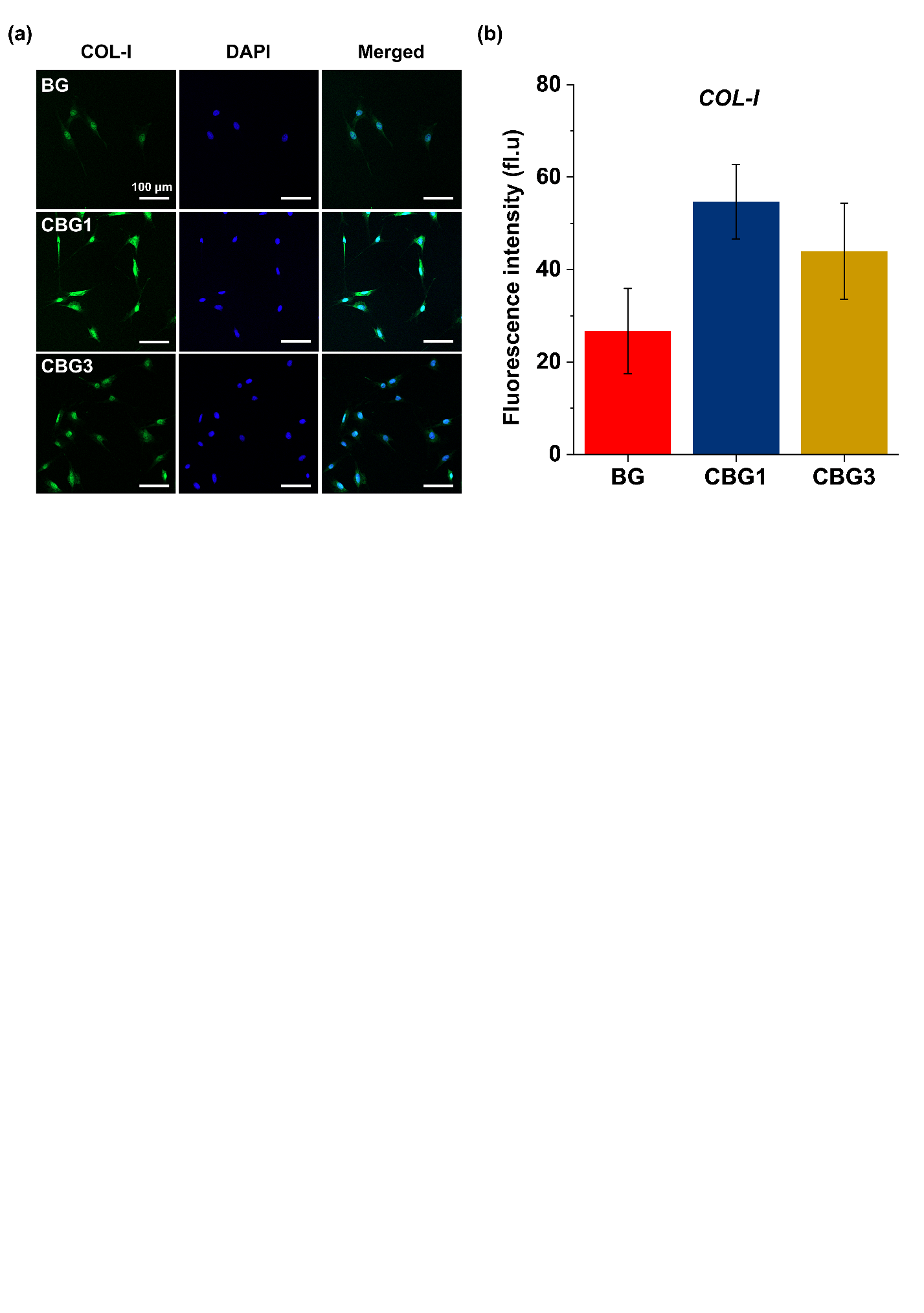


**Supplementary Figure 1.** Early osteogenic activity as COL-I marker of CBG groups against BG. (a)Representative immunocytochemistry images stained MC3T3-E1 (*n* = 3). (b) Measurement of fluorescence intensity (geometric mean). COL-I; type I collagen, BG; 45S5 Bioglass®, CBG; CeO_2_-doped BG.

**Supplementary Table 1. Energy dispersive spectrometry of BG and CBG groups**

Unit: weight%

| **Code** | **Si** | **Ca** | **P** | **Na** | **Ce** |
| --- | --- | --- | --- | --- | --- |
| BG | 41.50 | 30.26 | 8.02 | 20.22 | 0.00 |
| CBG1 | 41.52 | 32.72 | 7.75 | 16.26 | 1.76 |
| CBG3 | 42.93 | 28.02 | 7.92 | 17.28 | 3.85 |
| CBG6 | 42.83 | 29.09 | 8.04 | 10.73 | 9.31 |
| CBG12 | 39.99 | 26.27 | 7.02 | 10.32 | 16.40 |

Si; silicon, Ca; calcium, P; phosphorous, Na; sodium, Ce; cerium, BG; 45S5 Bioglass®, CBG; CeO_2_-doped BG.

The CBG groups were detected to the Si, Ca, P, Na, and Ce by energy dispersive spectrometry. The Ce element was consistent with the composition of CBG groups, with Ce levels augmentation.

**Supplementary Table 2. pH measurement of BG and CBG groups for 48 h**

| **Code** | **1 h** | **3 h** | **6 h** | **24 h** | **48 h** |
| --- | --- | --- | --- | --- | --- |
| BG | 8.05 ± 0.06^a^ | 8.30 ± 0.07^a^ | 8.42 ± 0.10^a^ | 8.41 ± 0.08^a^ | 8.40 ± 0.06^a^ |
| CBG1 | 8.04 ± 0.08^ab^ | 8.24 ± 0.05^ab^ | 8.44 ± 0.05^a^ | 8.46 ± 0.04^a^ | 8.47 ± 0.07^a^ |
| CBG3 | 8.01 ± 0.03^ab^ | 8.09 ± 0.02^bc^ | 8.18 ± 0.02^b^ | 8.22 ± 0.03^b^ | 8.21 ± 0.07^b^ |
| CBG6 | 7.91 ± 0.05^b^ | 7.99 ± 0.07^cd^ | 8.08 ± 0.04^b^ | 8.10 ± 0.05^bc^ | 8.08 ± 0.06^bc^ |
| CBG12 | 7.75 ± 0.06^c^ | 7.88 ± 0.04^e^ | 7.88 ± 0.04^c^ | 8.00 ± 0.07^c^ | 8.04 ± 0.06^c^ |
| *P*-value | 6.6 × 10^-6^ | 5.0 × 10^-6^ | 1.2 × 10^-6^ | 8.8 × 10^-6^ | 1.7 × 10^-5^ |

BG; 45S5 Bioglass®, CBG; CeO_2_-doped BG

Data indicated the mean and standard deviation. The data conducted a one-way analysis of variance (ANOVA) with Tukey’s post hoc test at a confidence level of 95%. The same small letter was no significant difference

**Supplementary Table 3. pH measurement of BG and CBG groups from 72 h to 168 h**

| **Code** | **72 h** | **96 h** | **120 h** | **144 h** | **168 h** |
| --- | --- | --- | --- | --- | --- |
| BG | 8.43 ± 0.09^a^ | 8.45 ± 0.04^a^ | 8.49 ± 0.02^a^ | 8.43 ± 0.04^a^ | 8.45 ± 0.05^a^ |
| CBG1 | 8.43 ± 0.07^ab^ | 8.41 ± 0.07^a^ | 8.40 ± 0.10^ab^ | 8.40 ± 0.08^a^ | 8.42 ± 0.06^a^ |
| CBG3 | 8.20 ± 0.08^ab^ | 8.22 ± 0.10^b^ | 8.23 ± 0.08^bc^ | 8.23 ± 0.08^b^ | 8.18 ± 0.06^b^ |
| CBG6 | 8.11 ± 0.07^b^ | 8.16 ± 0.04^bc^ | 8.12 ± 0.07^cd^ | 8.12 ± 0.07^b^ | 8.09 ± 0.08^b^ |
| CBG12 | 8.04 ± 0.07^c^ | 8.01 ± 0.05^c^ | 8.01 ± 0.05^d^ | 8.01 ± 0.05^b^ | 8.02 ± 0.05^b^ |
| *P*-value | 2.5 × 10^-5^ | 2.9 × 10^-6^ | 4.1 × 10^-5^ | 2.5 × 10^-4^ | 1.8 × 10^-5^ |

BG; 45S5 Bioglass®, CBG; Ce-doped BG.

Data indicated the mean and standard deviation. The data conducted a one-way analysis of variance (ANOVA) with Tukey’s post hoc test at a confidence level of 95%. The same small letter was no significant difference.

**Supplementary Table 4. Energy dispersive spectrometry of BG and CBG groups after immersion in the simulated body fluid for 14 days**

Unit: weight%

| **Code** | **Si** | **Ca** | **P** | **Na** | **Ce** |
| --- | --- | --- | --- | --- | --- |
| BG | 81.69 | 8.57 | 9.36 | 0.39 | 0.00 |
| CBG1 | 41.10 | 31.99 | 25.00 | 0.91 | 1.00 |
| CBG3 | 58.92 | 19.84 | 17.18 | 1.88 | 2.18 |
| CBG6 | 55.72 | 15.84 | 9.87 | 5.96 | 12.61 |
| CBG12 | 49.74 | 3.53 | 11.66 | 14.27 | 20.80 |

Si; silicon, Ca; calcium, P; phosphorous, Na; sodium, Ce; cerium, BG; 45S5 Bioglass®, CBG; CeO_2_-doped BG.
